# Left upper lung cancer with persistent left superior vena cava and left azygos vein: a case report

**DOI:** 10.1186/s13019-020-01278-w

**Published:** 2020-09-14

**Authors:** Zhongben Tang, Yin Teng, Jian Li, Xiaojun Du, Jiarong Xiao, Gongshun Tang

**Affiliations:** 1 Department of Thoracic, The Affiliated Hospital of Guizhou Medical University, 28 Guiyi Street, Guiyang, 550004 Guizhou China; 2 Department of Imaging, West China Hospital Sichuan University, 37 Guoxuexiang Street, Wuhou District, Chengdu, 610041 China

**Keywords:** Lung cancer, Persistent left superior vena cava, Left azygos vein, Surgery

## Abstract

**Background:**

With the popularization of thoracoscopic surgery, more and more macrovascular malformations have been reported. Understanding some vascular malformations with relatively fixed anatomical site and their range of drainage could avoid severe complications during the surgery. Persistent left superior vena cava (PLSVC) is a common thoracic vascular malformation, and is always combined with other cardiovascular dysplasia. As for the patient with upper left lung cancer in this case, he had PLSVC and left azygos vein, and non-metastatic enlargement of the lymph nodes at the same time, which had influenced the decisions on surgery and treatment. We made a summary of experience regarding this.

**Case presentation:**

A 46-years-old male patient, his CT found a space-occupying lesion in the superior lobe of the left lung. The chest CT showed that the patient had PLSVC and left azygos vein, and multiple enlarged lymph nodes in the mediastinum. The patient received thoracoscopic upper left lung lobectomy and lymph node dissection. It was discovered that the left azygos vein had a concealed form, which influenced the lymph node dissection. The post-surgery pathology showed that there was squamous cell carcinoma in the upper left lung (pT2bN0M0 p Phase IIA) and no cancer metastasis with the lymph nodes. The patient had a good post-surgery recovery.

**Conclusions:**

PLSVC is not rare, and is always combined with other vascular malformations. If discovering PLSVC before surgery, we suggest completing chest enhanced CT and vascular reconstruction, to find out other cardiovascular malformations that may exist. Left azygos vein is a rare vascular malformation, but it has a relatively fixed anatomical site, and always co-exists with PLSVC, therefore, understanding anatomy of left azygos vein is good for preventing accidental damage. Especially when performing surgery above the left pulmonary artery trunk, attention shall be paid to preventing damage to the left azygos vein. In addition, as for the patient with the diagnosis of lung cancer before surgery, it is not reliable to judge whether there is metastasis or not merely according to the size of the lymph nodes, instead, PET-CT or needle biopsy is recommended.

## Background

Persistent left superior vena cava (PLSVC) is formed due to non-degradation of the left superior vena cava after birth as a result of abnormal embryonic development. It is reported that, the incidence of PLSVC is about 0.3% ~ 0.5%, and some patient may have congenital heart disease or other vascular malformations [1]. PLSVC is one of the common variations in the thoracic vein system, while there is rarely no report on left azygos vein [2–6]. At present, there is no report on combination of such vascular malformations as well as radical surgery for left lung cancer. As for the patient in this case, he had a combination of two vascular malformations, and received upper left pneumonectomy and lymph node dissection. During the surgery process, we found that these malformed vessels especially left azygos vein had a concealed form, which could easily lead to accidental damage, thus massive hemorrhage. Therefore, we have summarized some previous experience regarding such vascular malformations.

## Case presentation

**Fig. 1:**
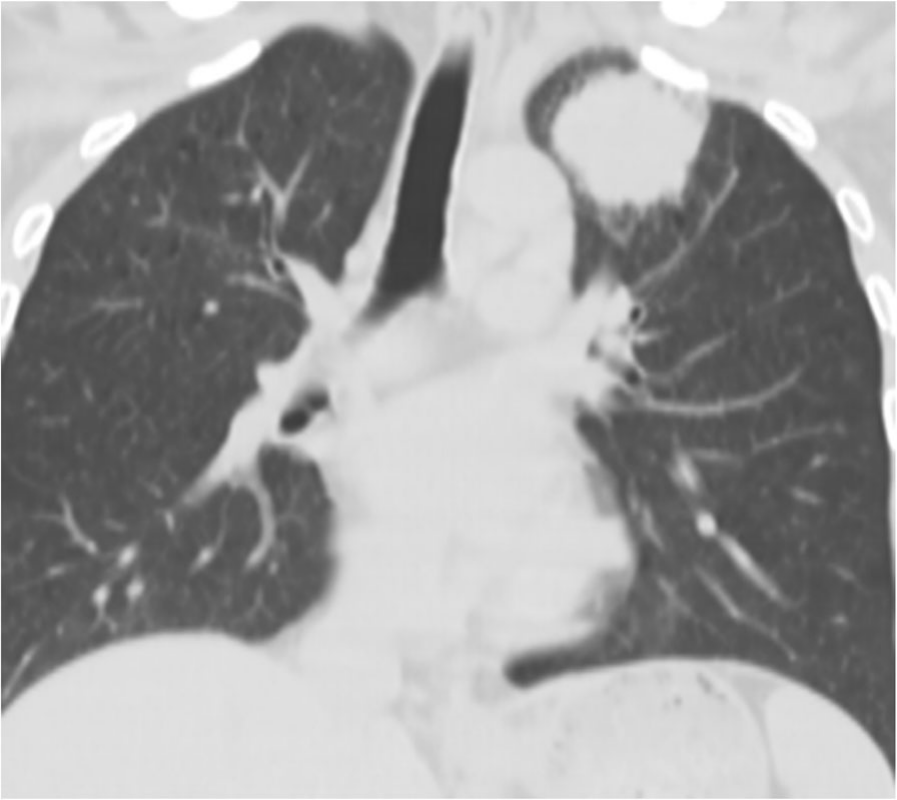
Left upper lung tumor

**Fig. 2:**
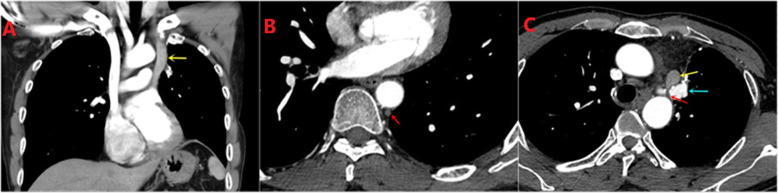
Persistent left superior vena cava(PLSVC) and left azygos vein (Chest CT which was shot 8 months after surgery). The left azygos vein is not easy to be detected before surgery.(red arrow: left azygos vein, yellow arrow: PLSVC, blue arrow: left pulmonary artery trunk)

A 46-years-old male patient, he was admitted to hospital due to chest pain for 2 weeks, and his CT found a space-occupying lesion in the superior lobe of the left lung (5.7*5.4*5.3 cm) (Fig. 1). The lesion was diagnosed as non-small-cell lung carcinoma according to pre-surgery percutaneous lung biopsy. The chest CT showed that the patient had PLSVC and left azygos vein (Fig. 2), and multiple enlarged lymph nodes in the mediastinum. The ultrasonic cardiogram considered PLSVC. Electrocardiogram and other examinations discovered no obvious abnormalities. The patient has a smoking history of 30 years, with 20 cigarettes per day, but he has no history of other diseases.

**Fig. 3:**
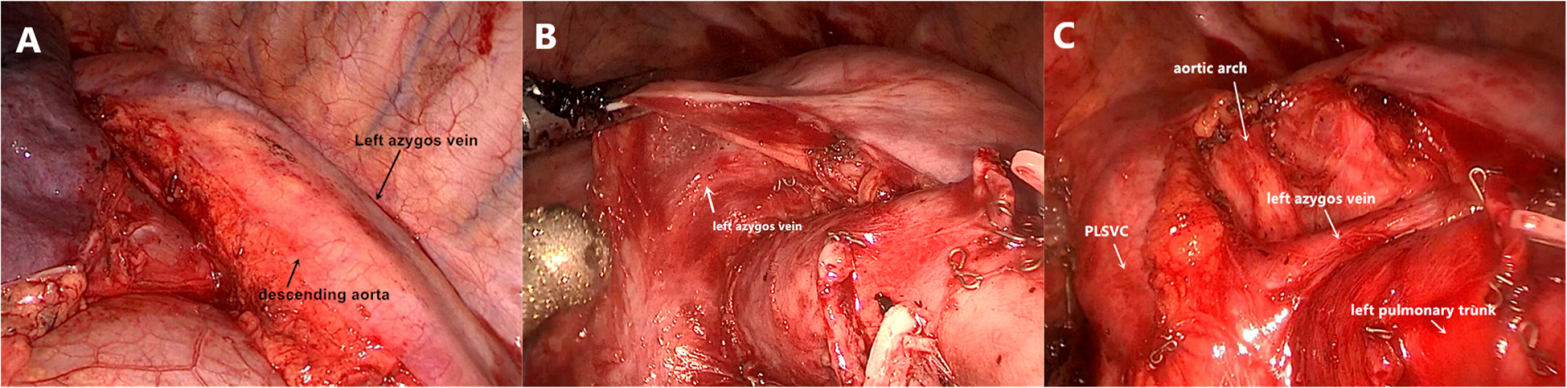
Left azygos vein

**Fig. 4:**
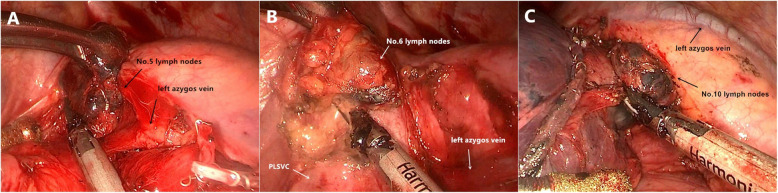
Lymph nodes of Group 5,6 and 10. The maximum diameter of lymph nodes of Group 5 was 2.5 cm

In January 2019, the patient received thoracoscopic upper left lung lobectomy and lymph node dissection. In the surgery, it was found that PLSVC stretched downward from the top of the chest, and abutted on the left margin of the arcus aortae and the ascending aorta, and then entered the pericardium at last. The left azygos vein entered the thoracic cavity via aortic hiatus, ascended along with the descending aorta, turned to the front at the intersection between the arcus aortae and the descending aorta, came to the deep connective tissues, went across the pulmonary artery trunk, and came to PLSVC at last (Fig. 3). There was no obvious malformation with the vein, artery and bronchus of the upper left lung. In the surgery, it could be seen that there was obvious enlargement of lymph nodes of the Group 4, 5, 6, 7 and 10, and the maximum diameter of single lymph node was about 2.5 cm (Fig. 4). Post-surgery pathology showed that: there was squamous cell carcinoma in the upper left lung (5*4*3 cm), reactive hyperplasia with the lymph nodes, and no invasion of the vessels, nerves, pleura or bronchial stump (pT2bN0M0 p Phase IIA). The patient received 6 chemotherapies after surgery. During a one-year follow-up visit, the patient had a good recovery without relapse.

## Discussion

PLSVC is a relatively common thoracic vascular malformation, and could be mostly diagnosed by chest CT, but there are also reports mistaking PLSVC as enlarged lymph nodes [7–9]. Most of PLSVC flow into the right atrium, while only a few into the left atrium. As for the patients with PLSVC into the right atrium, if without other cardiovascular malformations, they are always symptomless and require no therapy. However, as for those into the left atrium, they always demonstrate cyanosis of different degrees, and some require surgical treatment. At present, there are few reports regarding left lung cancer surgery combined with PLSVC [10], and some PLSVC patient may have other cardiovascular malformations at the same time [11], therefore, it needs to be extremely careful when performing left lung cancer surgery for such patients. In normal cases, some obvious cardiovascular malformations could be discovered by cardiac B-mode ultrasound and chest CT before surgery. In case of discovering PLSVC in pre-surgery examination, we suggest perfecting the vascular reconstruction in a further way, and reading the images carefully, so as to understand whether there are other vascular malformations or not, thus avoiding damage to these vessels during the surgery.

The patient in this case had PLSVC and left azygos vein at the same time. At present, there are very few reports on left azygos vein, but these reports all show that left azygos vein and PLSVC coexist with each other, and have a relatively fixed anatomical site. As for the patient in this case, since the left azygos vein had small caliber and concealed form, we didn’t notice the left azygos vein being purple on the surface of the descending aorta before surgery, and it was not noticed until during the surgery. However, at the intersection of the arcus aortae and the descending aorta, the left azygos vein bended forward, and flew into PLSVC crossing the left pulmonary artery trunk on deep surface of the connective tissues. Here, since left azygos vein was deep and not easy to observe, therefore, it was likely to damage the blood vessel and result in massive hemorrhage if not careful. The surgeon might mistake that the left pulmonary artery trunk or the arcus aortae was damaged. And for unexperienced surgeons, they may also mistake this as the arterial ligament. Therefore, regarding the patients with left azygos vein, when dissecting lymph nodes of the Group 5 and Group 6 and separating branches of the upper left lung artery, attention shall be paid to prevent damage to such vessel.

In addition, the patient had multiple enlarged lymph nodes in the mediastinum, and the maximum diameter of single lymph node was 2.5 cm. By experience, we were highly suspicious that the lymph node was metastatic lymph node, and combined with the tumor size, it was considered very likely to be Phase IIIb, and therefore, the patient might have no surgical indications. We suggested that the patient should perfect the PET-CT examination to know whether there was metastasis or not. However, the patient refused to complete PET-CT due to limitation of economic conditions, so we performed surgical treatment directly. Luckily, post-surgery pathology of the patient showed that there was no cancer metastasis with the lymph nodes. Therefore, we think that we can’t judge whether there is metastasis or not merely according to the size of lymph nodes, which might make the patients lose the opportunity for radical cure by surgery.

## Conclusion

To sum up, if discovering PLSVC before surgery, it generally means that there may be other vascular variations. We suggest completing chest enhanced CT and vascular reconstruction to understand whether there are other cardiovascular malformations or not. As for such vascular malformations as left azygos vein, at present, the reports all show that it coexists with PLSVC, and its anatomical site is relatively fixed. Therefore, being familiar with anatomy of left azygos vein is good for preventing accidental damage to the blood vessel, and especially when performing surgical operation above the left pulmonary artery trunk, extreme attention shall be paid. In addition, as for the patients with the diagnosis of lung cancer before surgery, it is not reliable to judge whether there is metastasis or not merely according to the size of the lymph nodes, instead, PET-CT or needle biopsy is recommended.

## Data Availability

All data generated or analyzed during this study are included in this published article.

## Abbreviations

PLSVC: Persistent left superior vena cava
PET-CT: Positron Emission Tomography-Computed Tomography
